# *O*-GlcNAc transferase regulates centriole behavior and intraflagellar transport to promote ciliogenesis

**DOI:** 10.1007/s13238-020-00746-2

**Published:** 2020-06-30

**Authors:** Fan Yu, Te Li, Yanchao Sui, Qingxia Chen, Song Yang, Jia Yang, Renjie Hong, Dengwen Li, Xiumin Yan, Wei Zhao, Xueliang Zhu, Jun Zhou

**Affiliations:** 1 Department of Genetics and Cell Biology, College of Life Sciences, State Key Laboratory of Medicinal Chemical Biology, Tianjin Key Laboratory of Protein Science, Key Laboratory of Bioactive Materials of the Ministry of Education, Nankai University, Tianjin, 300071 China; 2 College of Pharmacy, State Key Laboratory of Medicinal Chemical Biology, Nankai University, Tianjin, 300071 China; 3 State Key Laboratory of Cell Biology, Shanghai Institute of Biochemistry and Cell Biology, Center for Excellence in Molecular Cell Science, Chinese Academy of Sciences, Shanghai, 200031 China; 4 University of Chinese Academy of Sciences, Beijing, 100049 China; 5 Institute of Biomedical Sciences, Shandong Provincial Key Laboratory of Animal Resistance Biology, Collaborative Innovation Center of Cell Biology in Universities of Shandong, College of Life Sciences, Shandong Normal University, Jinan, 250014 China

**Dear Editor,**

*O*-GlcNAcylation is a nutrient sensor that is particularly sensitive to environmental glucose (Hardiville and Hart, 2014). Glucose can be converted to UDP-GlcNAc through the hexosamine biosynthetic pathway, providing a substrate for *O*-GlcNAcylation. Two enzymes participate in this reversible modification, *O*-GlcNAc transferase (OGT), which adds a single GlcNAc residue to the serine/threonine sites of proteins, and *O*-GlcNAcase (OGA), which removes the residue (Yang and Qian, 2017). OGT is a highly conserved, single gene-encoded protein that is ubiquitously expressed in higher eukaryotes, and human OGT shares more than 65% sequence identity with its *Caenorhabditis elegans* and *Drosophila melanogaster* orthologs (Jinek et al., 2004). *O*-GlcNAcylation can influence protein conformation, activity, interaction, half-life, and subcellular localization.

Almost all functional proteins are present among the pool of *O*-GlcNAcylated proteins, including enzymes, structural proteins, and transcription factors. Accordingly, *O*-GlcNAcylation can regulate complex processes, such as the cell cycle and embryonic development (Yang and Qian, 2017). Dysregulation of *O*-GlcNAcylation has been implicated in a wide range of pathologies, including cancer, neurodegeneration, cardiovascular diseases, and diabetes. The level of *O*-GlcNAcylation is greatly dysregulated by the abnormal glucose metabolism in diabetic mice and patients (Brownlee, 2001). In addition, we have demonstrated that dysregulation of *O*-GlcNAcylation is related to diabetic complications due to defects in cilia (Yu et al., 2019), which are hairlike protrusions present on the surface of most mammalian cells. However, the molecular details regarding the role of OGT in cilium assembly are still unclear.

To investigate the function of OGT in cilium formation, we generated OGT haploinsufficient mice. Because *Ogt* is an X-linked gene and complete knockout of OGT is lethal in mice, we first obtained female *Ogt*^fl/+^*Cre*^+^ and *Ogt*^fl/+^*Cre*^−^ mice by crossing *Ogt*^fl/fl^ mice with *Ubc-Cre-ERT2* mice (Figs. 1A and S1A). We then obtained *Ogt*^+/−^ and *Ogt*^+/+^ mice through intraperitoneal injection of tamoxifen (Fig. 1B). Two months after tamoxifen-induced knockdown of OGT, we found that the levels of OGT and protein *O*-GlcNAcylation were significantly reduced in *Ogt*^+/−^ mice (Fig. S1B). We then examined the morphology of cilia in these OGT haploinsufficient mice. Immunostaining of primary cilia and motile cilia in different tissues revealed a number of defects in *Ogt*^+/−^ mice. For example, retinal photoreceptor cilia, which are modified primary cilia, were fewer and shorter in *Ogt*^+/−^ mouse eyes than in *Ogt*^+/+^ mouse eyes; ciliary length reduced from ~1.5 μm to ~1.0 μm (Fig. 1C–E). In addition, we found fewer and shorter motile cilia in *Ogt*^+/−^ mouse trachea (Fig. 1F–I). Line profiles along the arrow-indicated regions revealed that OGT knockdown caused a significant decrease in the fluorescence intensity of tracheal epithelial cilia (Fig. 1F and 1G), and quantification revealed a ~50% decrease in the number of ciliated cells in the trachea (Fig. 1H). These data suggest that OGT is required for the formation of both primary cilia and motile cilia in mice.

**Figure 1:**
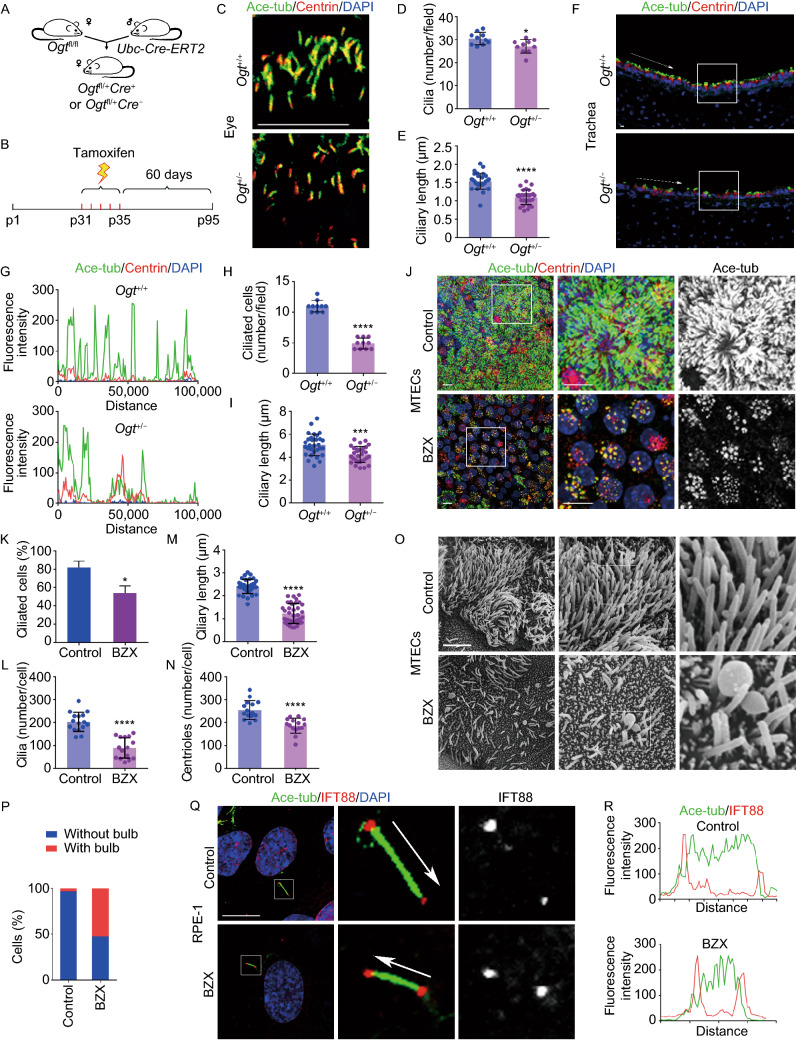
**OGT is required for cilium formation both in mice and in cells.** (A) *Ogt*^fl/fl^ mice were crossed with *Ubc-Cre-ERT2* mice to generate *Ogt*^fl/+^*Cre*^+^ and *Ogt*^fl/+^*Cre*^−^ mice. (B) Female *Ogt*^fl/+^*Cre*^+^ and *Ogt*^fl/+^*Cre*^−^ mice (P31) were administered tamoxifen daily by intraperitoneal injection for 5 consecutive days (P31–P35) and then normally bred for 60 days. *Ogt*^+/−^ and *Ogt*^+/+^ mice were obtained. (C–E) Eyes from *Ogt*^+/+^ and *Ogt*^+/−^ mice were subjected to immunofluorescence microscopy with antibodies against centrin and acetylated α-tubulin (C). Ciliary density (D, *n* = 10) and length (E, *n* = 30) were then quantified. (F–I) Trachea from *Ogt*^+/+^ and *Ogt*^+/−^ mice were subjected to immunofluorescence microscopy with antibodies against centrin and acetylated α-tubulin (F). The fluorescence intensity of acetylated α-tubulin in *Ogt*^+/+^ and *Ogt*^+/−^ mouse trachea was analyzed (G), along the arrows indicated in panel F. The number of ciliated cells per field (H, *n* = 10) and length (I, *n* = 30) were then quantified. (J and K) MTECs were cultured with BZX (50 μmol/L) from ALI day 3 to ALI day 9. Cells were immunostained with antibodies against centrin and acetylated α-tubulin and examined with confocal microscopy (J). The percentage of ciliated cells was then quantified (K, *n* = 100). (L–N) MTECs were cultured with BZX (50 μmol/L) from ALI day 3 to ALI day 9 and examined with SEM or 3D-SIM. The number of cilia per cell (L, *n* = 15), ciliary length (M, *n* = 30) and the number of centrioles per cell (N, *n* = 15) were quantified. (O and P) MTECs were cultured with BZX (50 μmol/L) from ALI day 3 to ALI day 9 and subjected to SEM (O). The percentage of cells containing cilia with or without bulbs was quantified (P, *n* = 100). (Q and R) RPE-1 cells were serum-starved, treated with BZX (150 μmol/L) for 48 h, and subjected to immunofluorescence microscopy with antibodies against IFT88 and acetylated α-tubulin (Q), and then the fluorescence intensity of IFT88 and acetylated α-tubulin from the basal body to the ciliary tip was quantified (R). Scale bars, 10 μm. *P < 0.05, ***P < 0.001, and ****P < 0.0001. Error bars indicate SD

We next investigated the role of OGT in the biogenesis of motile cilia using mouse tracheal epithelial cells (MTECs), which were cultured at an air-liquid interface (ALI) to induce the formation of multiple motile cilia. In contrast to cells with primary cilia, MTECs require a unique process involving the duplication of hundreds of centrioles, because each cilium requires a specialized centriole as a basal body (Zhao et al., 2013). After OGT activity was inhibited with a chemical compound containing a benzoxazolinone core (BZX) (Fig. S1C), which specifically inhibits OGT activity (Jiang et al., 2011), MTECs exhibited decreased ciliogenesis (Fig. 1J–N). The percentage of ciliated cells on ALI day 9 decreased from 80% to 50% (Fig. 1J and 1K). In addition, scanning electron microscopy (SEM) images revealed that few cilia protruded out of the membrane in BZX-treated cells (Fig. S1D), leading to fewer cilia per ciliated cell (Fig. 1L) and shorter cilia (Fig. 1M). Further investigation revealed an additional role of OGT in the unique duplication and scattering process of multiple centrioles. The basal bodies remained clustered in BZX-treated cells, in contrast to the even distribution of basal bodies in control cells (Fig. S1E and S1F). Moreover, the number of centrioles also decreased, implying impaired centriole duplication (Figs. S1E and 1N). These results were confirmed by OSMI-1, another OGT inhibitor (Fig. S1G and S1H). These results suggest that OGT regulates centriole behavior to promote ciliogenesis.

In addition to reduced ciliary number and length, we also found abnormal bulbs at the tips of cilia (Fig. 1O and 1P). These bulbs were clearly visible with SEM in both MTECs and human retinal pigment epithelial (RPE-1) cells (Figs. 1O and S2A). In MTECs with multicilia, the percentage of ciliated cells with bulbs was up to 50%; because there were hundreds of cilia in a single ciliated cell, a cell with even a single abnormal cilium was counted as a cell with a bulb (Fig. 1P). Upon BZX treatment, the percentage of ciliated RPE-1 cells with this abnormal feature increased from less than 5% to about 30% (Fig. S2A–C). The bulbs in RPE-1 cells were so prominent that they could also be easily detected under confocal microscopy with immunofluorescence staining (Fig. S2B). Because these structures were located at ciliary tips, we hypothesized that BZX treatment might compromise intraflagellar transport (IFT) along the axoneme of cilia. To test this possibility, we examined the localization of IFT proteins, including IFT88, which functions in anterograde transport in cilia and must be transported back by retrograde transport, and IFT140, which functions in retrograde transport. We found that OGT inhibition resulted in improper accumulation of IFT88 and IFT140 at the distal tips of cilia (Figs. 1Q, 1R, S2D and S2E). These data reveal a critical role for OGT in regulating the IFT process.

We then assessed the localization of OGT in different cell types, including MTECs and RPE-1, HeLa, MCF7, and U2-OS cells. In all cell types examined, OGT accumulated around the basal body/centrosome from which cilia protrude, suggesting that the enzyme may play a direct role in ciliogenesis (Figs. 2A and S3A). Because the centrosome structure varies widely during the cell cycle, U2-OS cells were synchronized to the G0/G1 phase after serum starvation to identify the precise localization of OGT. The cells were then labeled with centrosome markers and two different OGT antibodies and subjected to three-dimensional structured illumination microscopy (3D-SIM). Cross-sectional view of the centrosome revealed that the outer diameters of the OGT toroids were adjacent to cyclin-dependent kinase 5 regulatory subunit associated protein 2 (CDK5RAP2) and pericentrin (PCNT), which comprise the outer layer of the pericentriolar material (PCM). The two OGT antibodies corresponding to distinct regions of OGT showed slightly different sizes of the OGT toroids, implying a particular configuration of OGT in PCM (Fig. 2B–F). Co-staining of OGT with γ-tubulin showed both the cross-sectional view and radial direction localization of OGT (Fig. 2C). Cross-sectional view revealed that OGT formed a ring similar to that of γ-tubulin; however, the radial direction view showed that the localization of OGT was not exactly the same as that of γ-tubulin but slightly closer to the distal end of the centriole (Fig. 2C). Together, these data suggest that OGT is localized at the outer layer of the PCM.

**Figure 2:**
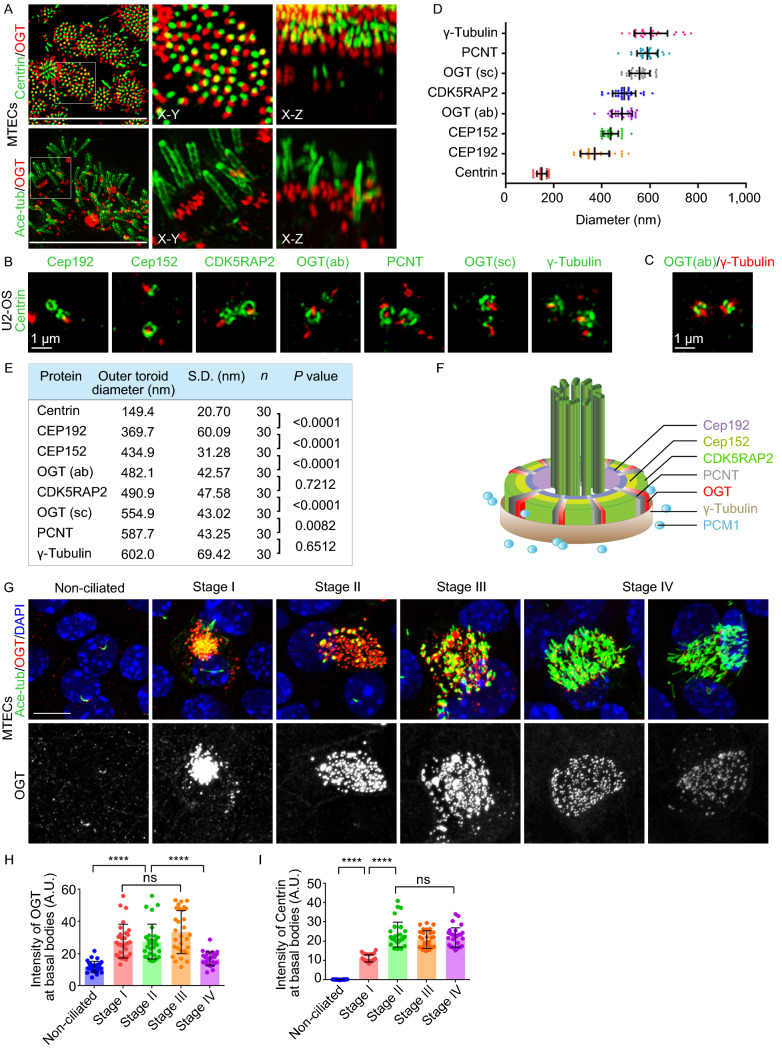
**OGT accumulates at the outer layer of the PCM and its localization changes during the ciliogenesis process.** (A) MTECs fixed on ALI day 5 were subjected to immunofluorescence microscopy with antibodies against OGT and acetylated α-tubulin or centrin. (B) U2-OS cells were serum-starved and subjected to 3D-SIM with antibodies against OGT or different centrosome markers and centrin. (C) U2-OS cells were subjected to 3D-SIM with antibodies against OGT and γ-tubulin. (D) Quantification of the outer toroid diameters (nm) of the indicated centrosome proteins. (E) Summary of the toroid quantifications measured in D. n represents the number of centrosomes measured. Mann-Whitney test was performed for statistical analysis. (F) Model for the organization of the centrosome proteins. (G–I) MTECs were subjected to immunofluorescence microscopy for OGT and acetylated α-tubulin and grouped at different stages (G). The intensity of OGT and centrin at the basal bodies grouped to the indicated stages was then quantified by Mann-Whitney test (H and I). Scale bars, 10 μm unless specified. ****P < 0.0001; ns, not significant. Error bars indicate SD

In agreement with our previous findings (Yu et al., 2019), we found that the localization of OGT at the basal body/centrosome was not static in RPE-1 cells. Upon the induction of ciliogenesis by serum starvation, there was a gradual decrease in OGT localization at the basal body/centrosome and an increase in OGT localization at the nucleus (Fig. S3B). The levels of acetylated α-tubulin and γ-tubulin showed no clear changes during ciliogenesis in RPE-1 cells (Fig. S3C). However, the level of OGT was slightly decreased during ciliogenesis, in accordance with a gradual increase in the level of *O*-GlcNAcylation (Fig. S3C).

Ciliogenesis is more complicated in multiciliated cells, because it involves an extra stage of robust centriole duplication (Zhao et al., 2013). As a result, in contrast to the observation in RPE-1 cells, the OGT level was significantly elevated as multiciliogenesis progressed, similar to other centrosomal markers (Fig. S3D). Interestingly, the level of OGT decreased at the end of multiciliogenesis (ALI day 11, Fig. S3D). This phenomenon was confirmed by immunofluorescence staining. OGT accumulated at the basal body along with centrin in MTECs. On ALI day 2 and ALI day 3, when centriole duplication began, OGT clusters emerged and kept gradually increasing. However, by ALI day 11, when cilia had almost reached full length, OGT localization at the basal bodies decreased (Fig. S3E). We then grouped MTECs into different stages. The first detectable sign of centriole formation was at stage I (began on ALI day 2); at this stage, foci of centrosomal markers and OGT appeared near the centrosome (Figs. 2G and S3E). From ALI day 3 to ALI day 11, cells in other stages appeared sequentially during the culture period. In stage II, centrosomal proteins began to localize to a single dense cluster. During stage III, centrioles dispersed from the cluster toward the plasma membrane. On ALI day 11, most cells reached stage IV; At stage IV, when axoneme formation began shortly after the centrioles reached the plasma membrane, OGT localization at the basal body decreased while centrin showed no such change (Figs. 2G–I and S3F). Together, these data suggest that OGT primarily functions at the early stage of ciliogenesis.

Post-translational modifications, such as acetylation, phosphorylation, ubiquitination, and polyglutamylation, are known to regulate ciliogenesis (Tang et al., 2013; Yang et al., 2014; Yu et al., 2016; Yang et al., 2019; Ran et al., 2020). In addition, our recent findings indicate that proper regulation of *O*-GlcNAcylation is also critical for cilium formation (Yu et al., 2019). In this study, we have focused on the role of OGT in ciliogenesis because of its particular localization at the basal body. OGT inhibition led to a number of ciliary defects, including fewer ciliated cells, shorter ciliary length, and the presence of abnormal bulbs at the tips of cilia. Similar to OGT, OGA also affected ciliogenesis, although to a lesser degree (data not shown), which we believe is most likely due to the feedback regulation between OGT and OGA.

OGT is composed of two separate domains. The C-terminal domain has glycotransferase activity while the N-terminal domain consists of multiple tetratricopeptide repeats (TPRs). The TPR domains interact with other proteins and are essential for OGT oligomerization (Jinek et al., 2004). The TPR domains are also responsible for the localization to the centrosome of certain proteins such as monopolar spindle 1 (Mps1) (Marquardt et al., 2016), trafficking protein particle complex 8 (TRAPPC8), -9, -10, and -11 (Schou et al., 2014), and human homolog of cell division control 27 (CDC27Hs) and CDC16Hs (Tugendreich et al., 1995).The present study reveals a delicate localization of OGT in the outer layer of the PCM. Further work is required to determine whether the TPR domains of OGT are responsible for its localization to the centrosome, especially the outer layer of the PCM.

In the present study, super-resolution microscopy images show that OGT is localized adjacent to PCNT at the PCM. Interestingly, OGT forms a wreath-like pattern around the centriole, just like PCNT and γ-tubulin. As an enzyme, this structural protein-like localization is certainly intriguing. Whether OGT plays a role in the construction of the centrosome architecture requires further exploration. In addition, because the main function of PCM is to anchor microtubules for subsequent transport of various proteins, it is tempting to speculate that OGT might participate in ciliogenesis by regulating protein transport to the centrosome and cilia. Further studies to identify centrosome- and cilium-related OGT substrates would allow verification of this hypothesis.

## Electronic supplementary material

Below is the link to the electronic supplementary material.

Supplementary material 1 (PDF 617 kb)
