# Supplementary material for: O-GlcNAc transferase regulates centriole behavior and intraflagellar transport to promote ciliogenesis
Source: Protein Cell. 2020 Jun 30;11(11):852–7. doi: 10.1007/s13238-020-00746-2 (PMC7647980; doi:10.1007/s13238-020-00746-2)
Supplement: Supplementary file 1 — Supplementary material 1 (PDF 617 kb) [file 13238_2020_746_MOESM1_ESM.pdf]

Materials and Methods

Mice

Ogt-floxed mice on a C57BL/6 background were obtained from the Jackson Laboratory (Bar Harbor, ME). *Ubc-Cre-ERT2* mice were kindly provided by Dr. Wen Ning (Nankai University). To obtain OGT haploinsufficient mice, 1-month-old $Ogt^{fl/+}Cre^{+}$ and $Ogt^{fl/+}Cre^{-}$ mice were administered tamoxifen (Sigma-Aldrich) in corn oil by daily intraperitoneal injection at 75 μg/g body weight for 5 consecutive days. Animal experiments were performed in accordance with protocols approved by the Animal Care and Use Committee of Nankai University.

Antibodies and chemicals

Antibodies against OGT (sc-32921), GAPDH (Santa Cruz Biotechnology), acetylated α-tubulin, β-actin, *O*-GlcNAc (Sigma-Aldrich), OGT (ab96718), α-tubulin, γ-tubulin, PCNT-N1 (Abcam), IFT88, IFT140, centrin (Proteintech), CEP152, CEP192, and CDK5RAP2 (Bethyl) were purchased from the indicated sources. Alexa Fluor 488 and 568 secondary antibodies were from Life Technologies, and DAPI was from Sigma-Aldrich. BZX was synthesized following the procedures described previously (Inhibitor 2 described in the reference) (Jiang et al., 2011). OSMI-1 (SML1621) was purchased from Sigma-Aldrich.

Cell culture

RPE-1 cells were obtained from the American Type Culture Collection and grown in DMEM/F12 medium supplemented with 10% fetal bovine serum. To induce cilium formation, RPE-1 cells were cultured in serum-free DMEM/F12 medium for 24 or 48 h. HeLa, U2-OS, and MCF7 cells were obtained from the American Type Culture Collection and grown in DMEM medium supplemented with 10% fetal bovine serum. MTECs were isolated from 6-week-old C57BL/6J mice and cultured as described previously (You et al., 2002).

Immunofluorescence microscopy

Mouse tissues were fixed in 4% paraformaldehyde, embedded in Tissue-Tek OCT (Sakura), and quick-frozen in liquid nitrogen. Thin sections were pre-permeabilized in 0.1% Triton X-100/phosphate-buffered saline (PBS) for 2 min, fixed with 4% paraformaldehyde for 30 min, and permeabilized in 0.5% Triton X-100/PBS for 25 min. The tissues were then blocked in 4% bovine serum albumin (BSA) for at least 1 h and stained with primary antibodies at 4°C overnight. They were then stained with fluorescein-conjugated secondary antibodies, followed by DAPI. The sections were subsequently mounted onto slides and examined with a Zeiss LSM710 confocal microscope. Cells grown on glass coverslips were fixed in ice-cold methanol for 3 min, blocked with 4% BSA, and then incubated with primary antibodies, secondary antibodies, and DAPI. MTECs on Transwell membranes were pre-extracted with 0.5% Triton X-100 in PBS for 3 min and then fixed with 4% fresh paraformaldehyde in PBS

for 15 min at room temperature. After fixation, the cells were permeabilized with 0.5% Triton X-100/PBS for 15 min and blocked with 4% BSA/PBS for 1 h. The cells were then incubated with antibodies and examined with confocal microscopy or 3D-SIM (GE DeltaVision OMX SR). The length of cilia, the percentage of ciliated cells, and the diameter of the centrosome toroid were measured with ImageJ (National Institutes of Health).

Electron microscopy

Transwell membranes with MTECs were isolated and fixed with 2.5% glutaraldehyde in 0.1 M PBS at 4°C overnight. Samples were post-fixed in 1% osmium tetroxide for 1 h, dehydrated in ethanol gradient buffer, and dried by critical point drying. The samples were then gold-coated by the sputter technique and examined with a QUANTA 200 scanning electron microscope (FEI) at an accelerating voltage of 15 kV.

Statistical analysis

All quantitative data are presented as the mean ± SD of at least three independent experiments and were compared by using the Student's *t*-test (no specific illustration) or Mann-Whitney test.

Acknowledgments

We thank Dr. Wen Ning for providing the Ubc-Cre-ERT2 mice. This work was supported by grants from the National Key R&D Program of China (2017YFA0503502) and the National Natural Science Foundation of China (31991193).

Compliance with Ethics Guidelines

Fan Yu, Te Li, Yanchao Sui, Qingxia Chen, Song Yang, Jia Yang, Renjie Hong, Dengwen Li, Xiumin Yan, Wei Zhao, Xueliang Zhu, and Jun Zhou declare that they have no conflict of interest. All institutional and national guidelines for the care and use of laboratory animals were followed.

Jiang, J., Lazarus, M.B., Pasquina, L., Sliz, P., and Walker, S. (2011). A neutral diphosphate mimic crosslinks the active site of human O-GlcNAc transferase. Nat Chem Biol 8, 72-77.

You, Y., Richer, E.J., Huang, T., and Brody, S.L. (2002). Growth and differentiation of mouse tracheal epithelial cells: selection of a proliferative population. Am J Physiol Lung Cell Mol Physiol 283, L1315-1321.

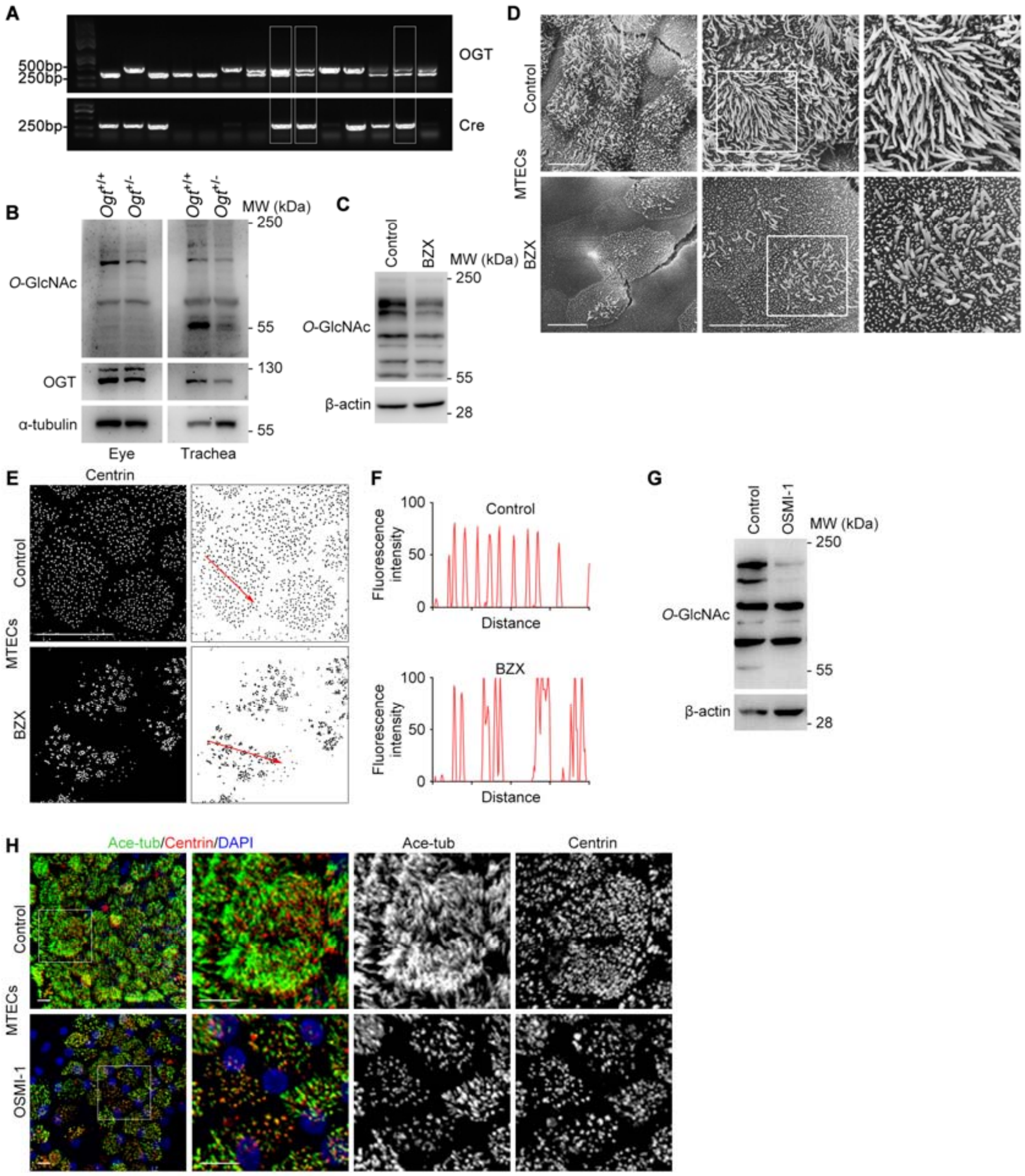

Fig. S1. (A) Genotyping by PCR with OGT and Cre primers to identify first-generation mice. PCR was performed using mouse-tail DNA from female offspring of *Ogt*$^{fl/fl}$ mice and *Ubc-Cre-ERT2* mice. (B) Eyes and trachea from *Ogt*$^{+/+}$ and *Ogt*$^{+/-}$ mice were subjected to immunoblotting. (C) MTECs were cultured with BZX (50 µM) from ALI day 3 to ALI day 9. Cells were subjected to immunoblotting with antibodies against *O*-GlcNAc and β-actin. (D-F) MTECs were cultured with BZX (50 µM) from ALI day 3 to ALI day 9. Cells were examined with SEM (D) or 3D-SIM (E). The fluorescence intensity of centrin along the arrows indicated in panel E was quantified (F). (G) MTECs were cultured with OSMI-1 (10 µM) from ALI day 3 to ALI day 9. Cells were subjected to immunoblotting with antibodies against *O*-GlcNAc and β-actin. (H) MTECs were cultured with OSMI-1 (10 µM) from ALI day 3 to ALI day 9. Cells were immunostained with antibodies against centrin and acetylated α-tubulin and examined with confocal microscopy. Scale bars, 10 µm.

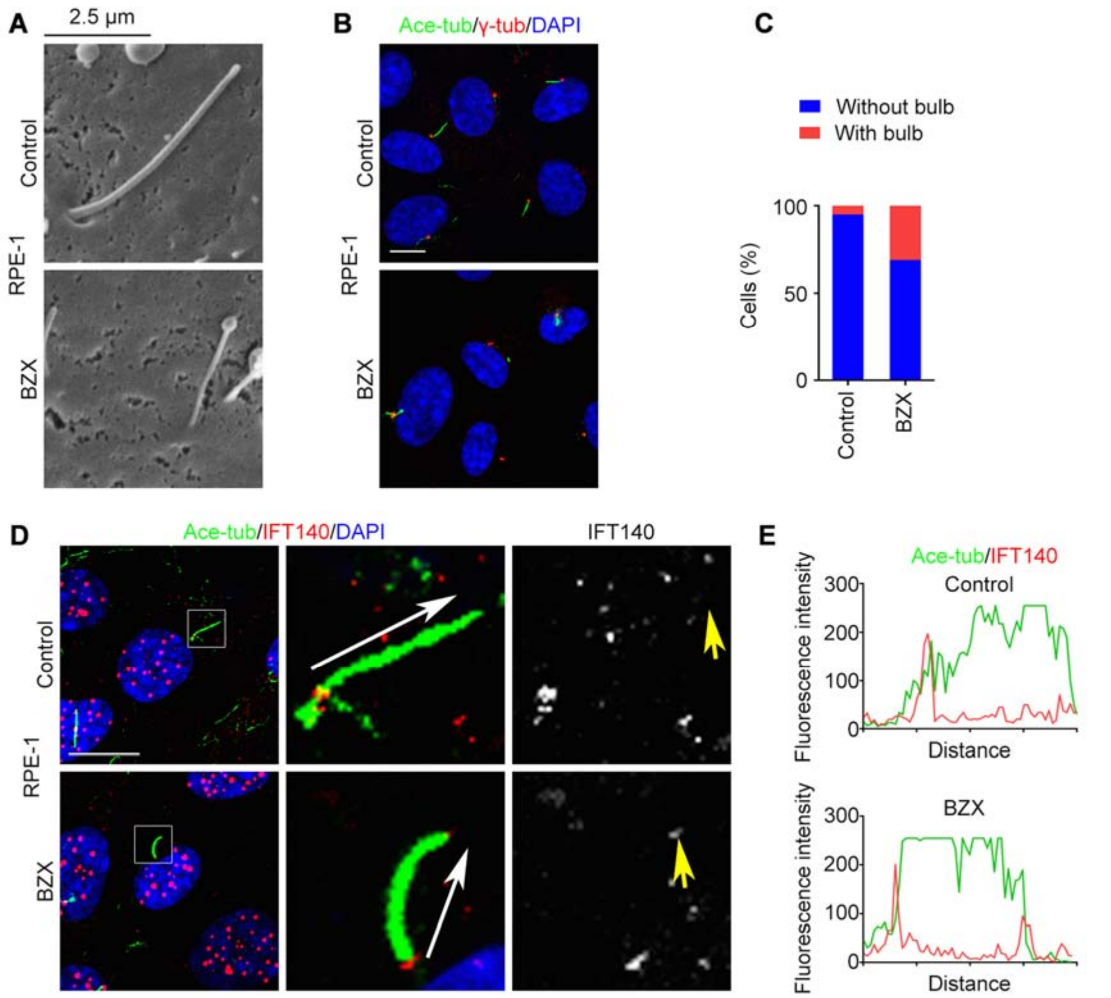

Fig. S2. OGT inhibition leads to the formation of bulbs at ciliary tips and impairs the IFT process. (A–C) RPE-1 cells were treated with BZX (150 μM) and serum-starved for 48 h. Cells were subjected to SEM (A) or immunostained with antibodies against γ-tubulin and acetylated α-tubulin (B). The percentage of ciliated cells with or without bulbs at the tips was quantified (C, n = 100). (D and F) RPE-1 cells were serum-starved, treated with BZX (150 μM) for 48 h, and subjected to immunofluorescence microscopy with antibodies against IFT140 and acetylated α-tubulin (D). The fluorescence intensity of IFT140 and acetylated α-tubulin from the basal body to the ciliary tip was quantified (E). Scale bars, 10 μm unless specified.

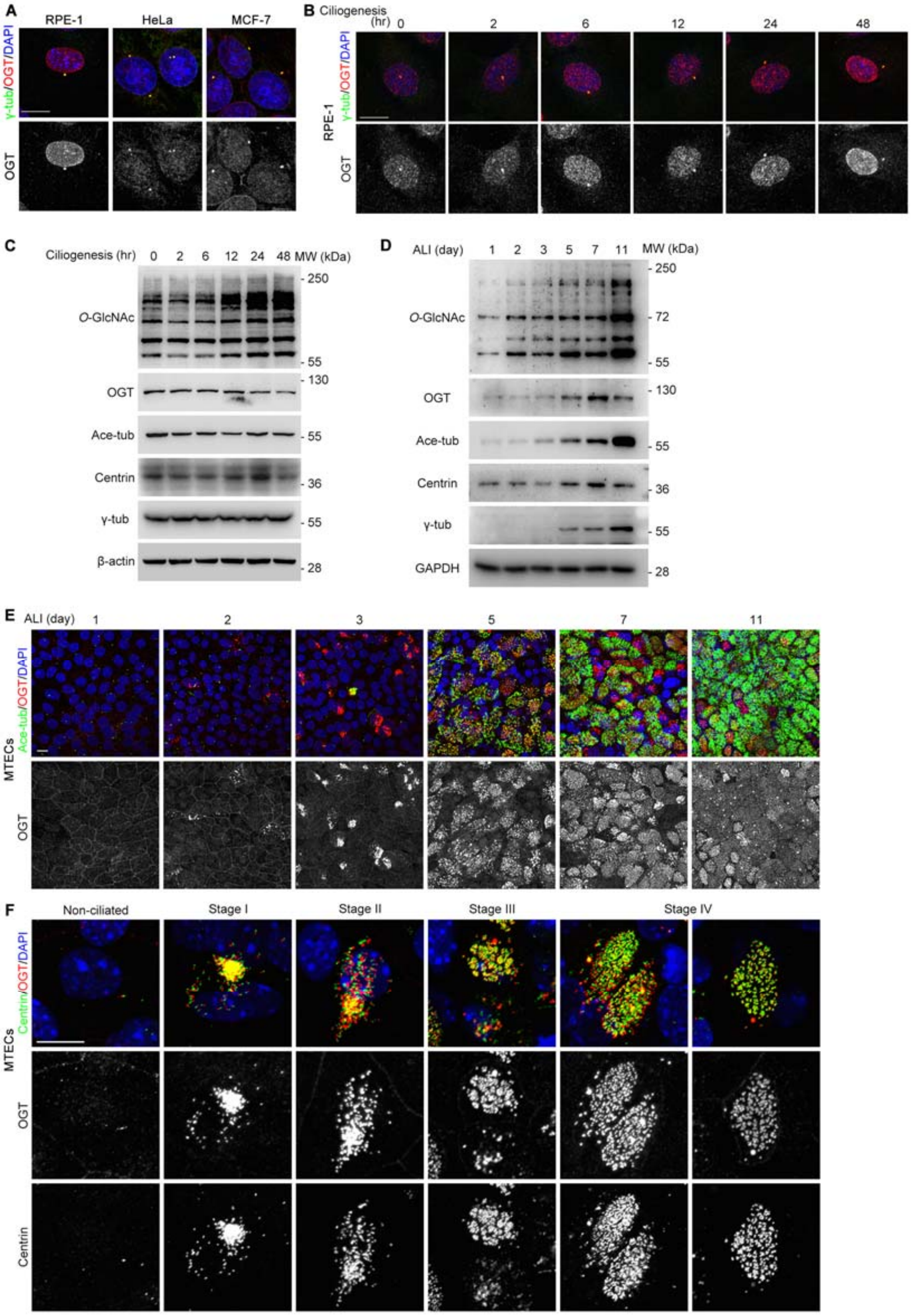

Fig. S3. (A) Cells were subjected to immunofluorescence microscopy with antibodies against OGT and γ-tubulin. (B) RPE-1 cells were serum-starved for the indicated time and immunostained with antibodies against OGT and γ-tubulin. (C) RPE-1 cells were serum-starved for the indicated time and subjected to immunoblotting for OGT and centrosome/cilium-related proteins. (D and E) MTECs at the indicated ALI days were subjected to immunoblotting (D) and immunofluorescence microscopy (E). (F) MTECs were subjected to immunofluorescence microscopy for OGT and centrin and grouped at different stages. Scale bars, 10 µm.
